# A time-course prediction model of global COVID-19 mortality

**DOI:** 10.3389/fpubh.2023.1232531

**Published:** 2023-12-07

**Authors:** Mark Ciaccio, Chris Schneiderman, Abhishek Pandey, Robert Fowler, Kevin Chiou, Gage Koeller, David Hallett, Whitney Krueger, Leon Raskin

**Affiliations:** ^1^AbbVie Inc., North Chicago, IL, United States; ^2^Sunnybrook Health Sciences Center, Toronto, ON, Canada; ^3^Meta Reality Labs, Burlingame, CA, United States; ^4^Tulane University School of Public Health and Tropical Medicine, New Orleans, LA, United States

**Keywords:** COVID-19, machine learning, mortality, partial least squares regression, elastic net

## Abstract

**Introduction:**

The COVID-19 pandemic has caused over 6 million deaths worldwide and is a significant cause of mortality. Mortality dynamics vary significantly by country due to pathogen, host, social and environmental factors, in addition to vaccination and treatments. However, there is limited data on the relative contribution of different explanatory variables, which may explain changes in mortality over time. We, therefore, created a predictive model using orthogonal machine learning techniques to attempt to quantify the contribution of static and dynamic variables over time.

**Methods:**

A model was created using Partial Least Squares Regression trained on data from 2020 to rank order the significance and effect size of static variables on mortality per country. This model enables the prediction of mortality levels for countries based on demographics alone. Partial Least Squares Regression was then used to quantify how dynamic variables, including weather and non-pharmaceutical interventions, contributed to the overall mortality in 2020. Finally, mortality levels for the first 60 days of 2021 were predicted using rolling-window Elastic Net regression.

**Results:**

This model allowed prediction of deaths per day and quantification of the degree of influence of included variables, accounting for timing of occurrence or implementation. We found that the most parsimonious model could be reduced to six variables; three policy-related variables – COVID-19 testing policy, canceled public events policy, workplace closing policy; in addition to three environmental variables – maximum temperature per day, minimum temperature per day, and the dewpoint temperature per day.

**Conclusion:**

Country and population-level static and dynamic variables can be used to predict COVID-19 mortality, providing an example of how broad temporal data can inform a preparation and mitigation strategy for both COVID-19 and future pandemics and assist decision-makers by identifying population-level contributors, including interventions, that have the greatest influence in mitigating mortality, and optimizing the health and safety of populations.

## Introduction

The COVID-19 pandemic is among the most disastrous in history and the first large pandemic in the last 100 years. COVID-19, which is caused by SARS-CoV-2, is not the first pandemic originating from the Coronaviridae family; both severe acute respiratory syndrome (SARS) and Middle East respiratory syndrome (MERS) were caused by newly identified coronaviruses (1). The SARS pandemic began in the Guangdong Province in China (2002–2004) with a mortality rate of 10%, around 8,000 infections, and nearly 750 deaths (2). The MERS pandemic began in Saudi Arabia in 2012 before spreading to 27 different countries with around 2,604 confirmed cases, 936 deaths, and an mortality rate of 36% (3). COVID-19 had a global impact, affecting almost all countries in the world in 2020–2023 and leading to at least 7 M – 20 M deaths thus far (4, 5).

With the COVID-related data reported waning, and experiencing significant data issues (4), many models have discontinued producing forecasts altogether (5–7).

Non-Pharmaceutical Interventions (NPIs) including workplace closings, stay-at-home orders, quarantine measures, mask mandates, social distancing standards, and the closing of public events were implemented in most countries (8). The efficacy of these interventions are still debated and may vary considerably by country, demographic and societal factors, and likely climate differences across regions. Therefore, knowing how country demographics, weather patterns, and NPIs may have contributed to COVID-19 mortality is important when investigating mechanisms to minimize the COVID-19 death toll as well as to provide data-driven solutions to mitigate mortality from future pandemics.

Machine learning can facilitate our understanding of factors which contribute to covid mortality by incorporating simultaneously static and dynamic variables in a single predictive model. The object of this study is the evaluation of the contribution and directionality of each variable relative to each other using machine learning methods. Specifically, we (1) rank order the significance and estimate effect size of explanatory variables and their contribution to COVID-19 mortality, through implementation of two machine learning methods, Partial Least Squares Regression (PLSR) and sliding-window Elastic Net Regression (ENR); and (2), create time-course predictions for COVID-19 mortality, by country. This data-driven approach can then be used to inform the responses, in accordance with the importance of each driver, with a goal of mitigating mortality from COVID-19.

## Materials and methods

### Rationale

Several manuscripts have previously used machine learning to predict COVID-19 mortality (9–11). We refer readers to the following reviews to highlight the strengths of weaknesses of various approaches: Every predictive algorithm has strengths and weaknesses due to inherent trade-offs between accuracy, speed, and interpretability. In this manuscript, we used two methods (PLSR and sliding-window Elastic Net regression). Both methods were chosen to strike a balance between being easily interpretable, having the speed to be updated in seconds, and having the accuracy to give actionable daily predictions for countries across the globe. The strength of this analysis is the ability to give actionable advice to global corporations, governments, and agencies. The predictions facilitate providing sufficient resources by country to prepare ahead of spikes in mortality as well as provide actionable interventions to decrease the future global burden of the pandemic.

## Strengths and limitations

Both algorithms (PLSR and sliding-window Elastic Net regression) use the assumptions of independent and identically distributed random normal variables. In addition, the linear models assume additive effects of each variable to the response. These assumptions therefore may have less predictive accuracy using variables such as alcohol consumption by country which does not follow a normal distribution or miss complex interactions arising between the variable interactions. In addition, PLSR assumes that the response is generated by a process that is driven a small number of latent (not directly observed) variables.

Strengths of these models is their ability to account for a high amount of collinearity in the system. Almost all predictive variables used in this study have some correlation to each other. The Elastic Net and PLSR both control well for the information that may be redundant between variables, increasing the accuracy of predictions on data outside the training set, and reducing the wide array of variables in the dataset to a reduced set of factors. This allows the model to be simplified for accuracy, speed, and particularly interpretation. If the system can be accurately predicted with a small amount of inputs, one can assume that the causal factors driving regional mortality may be small enough to be identified and controlled.

### Data acquisition

Static and dynamic explanatory and response/outcome variables were acquired from a wide array of data sources for each country with available daily mortality data (Table 1). Mortality during calendar year 2020 was acquired from John Hopkins Coronavirus Resource Center (6, 12). Static variables providing information of a countries general health and healthcare situation prior to any experience with COVID-19 (2018–2019) were acquired from Kaggle, the US Central Intelligence Agency (CIA), World Bank (WB), Oxford University’s Our World In Data platform (OWID) and the World Health Organization (WHO) (13–17). Dynamic variables which characterize each country’s response to COVID-19 during the pandemic were acquired from Oxford’s COVID-19 Government Response Tracker, and daily climate measures from the National Oceanic and Atmospheric Administration (NOAA) for calendar year 2020 (18, 19).

**Table 1:** Source of static and dynamic variable data chosen for the predictive model.

Variable type	Description	Source
Outcome variable		
Daily reported COVID-19 mortality	Mortality by country, by day, 14 day moving average	John Hopkins (6, 12)
Static variables		
Alcohol consumption	Proportion of daily energy derived from alcohol consumption	Kaggle (13)
Median age	Median age by country	CIA (14)
Obesity	Percent BMI ≥ 30 – age 18+	WHO (22)
DALYs – HIV/AIDS and tuberculosis	DALYs related to HIV/TB	OWID (16)
Percent Urban	Percent of country population in urban areas	WB (15)
DALYs – Cardiovascular diseases	DALYs related to CV diseases	OWID (16)
DALYs – Chronic respiratory diseases	DALYs related to respiratory diseases	OWID (16)
DALYs – Diabetes, urogenital, blood, and endocrine diseases	DALYs related to diabetes, etc.	OWID (16)
DALYs – Self-harm	DALYs related to self-harm	OWID (16)
DALYs – Interpersonal violence	DALYs related to interpersonal violence	OWID (16)
Child immunization (DTP3)	Percentage of infants receiving three doses of diphtheria-tetanus-pertussis– containing vaccine (DTP3)	WHO (17)
At least basic sanitation	Percentage of households using at least basic sanitation facilities	WHO (17)
Mean fasting plasma glucose	Age-standardized mean fasting plasma glucose for adults aged 18 years and older	WHO (17)
Tobacco nonsmoking	Age-standardized prevalence of adults aged 15 years and older not smoking tobacco in last 30 days	WHO (17)
Hospital bed density	Hospital beds *per capita*, relative to a maximum threshold of 18 per 10,000 population	WHO (17)
Health worker density	Health professionals (physicians, psychiatrists and surgeons) *per capita*, relative to maximum thresholds for each cadre	WHO (17)
International health regulations core capacity index	Average percentage of attributes of 13 core capacities	WHO (17)
Dynamic variables (time dependent)		
Daily minimum and maximum temperature	Minimum / maximum Daily Temp	NOAA (19)
Percent of day with precipitation	Hourly measure of precipitation	NOAA (19)
Dewpoint temperature per day	Dewpoint per day	NOAA (19)
Cancelled Public Events policy	Record cancelling public events	Oxford (18)
Restrictions on gatherings policy	Record limits on gatherings	Oxford (18)
Movement restriction policy	Record restrictions on internal movement between cities/regions	Oxford (18)
Face coverings – masking policy	Record policies on the use of facial coverings outside the home	Oxford (18)
Shelter in place policy	Record orders to “shelter-in-place” and otherwise confine to the home	Oxford (18)
Income support policy	Record if the government is providing direct cash payments to people who lose their jobs or cannot work.	Oxford (18)
Contract tracing policy	Record government policy on contact tracing after a positive diagnosis	Oxford (18)
School closing policy	Record closings of schools and universities	Oxford (18)
Public transit closing policy	Record closing of public transport	Oxford (18)
Public information campaigns	Record presence of public info campaigns	Oxford (18)
International travel policy	Record restrictions on international travel	Oxford (18)
COVID-19 Testing policy	Record government policy on who has access to testing	Oxford (18)
Debt relief policy	Record if the government is freezing financial obligations for households (e.g., stopping loan repayments, preventing services like water from stopping, or banning evictions)	Oxford (18)
Workplace closing policy	Record closings of workplaces	Oxford (18)

### Data preparation

All analysis was performed using Python 3.8 on a Cloudera Machine Learning environment using 8 CPUs and 32 GiB memory. All visualization including heatmaps, clustergrams, scatterplots, bar graphs, and line graphs were created using Python packages matplotlib-3.1.3, adjustText-0.7.3, and seaborn-0.11.2. For heatmaps, rows were min-max normalized between 0–1 for clarity.

Daily deaths by country were calculated by subtracting the prior days cumulative mortality from the current day cumulative. Daily deaths then were converted to 14-day moving averages for each country. Countries which had fewer than 200 deaths during 01 Jan 2020 through 31 Dec 2020 were excluded from the analysis. Weather was mapped to respective countries using latitude and longitude from the NOAA data lake. Weather information including temperatures, dew point, and days with precipitation was averaged for every weather station reported within the area of a country. To acquire an average of weather patterns, data was used from the years 2010–2020 and averaged for all years before analysis. Any given variable for which more than 5 years of data was missing was removed from the analysis. Climate measures that had more than five years of missing data for a given variable were removed from the analysis. All data was manually downloaded and stored as comma-separated value files from respective sources. The data was merged using the ISO3 country codes. Missing data was imputed using the median values for the respective variable from all other countries.

### Statistical modeling

To cluster countries by mortality dynamics, we min-max normalized the daily mortality values by country and used cosine similarity. We manually chose six clusters based on the hierarchical clustering to help conceptualize countries with similar COVID-19 dynamics. The clusters were used for visualization only; all countries with more than 200 reported deaths within 2020 were used separately to train the predictive models.

Using partial least squares regression, all explanatory variables were unit normalized to have a mean of 0 and a standard deviation of one before modelling. The PLSRegression() function was used from the scikit-learn-0.24.2 python package. Model accuracy over a range of principal components was assessed using the root mean squared error (RMSE). The number of principal components to use in the final model was chosen using the elbow rule (20). Two principal components were used in the static variable model and four for the dynamic model.

To train the machine learning algorithm to predict COVID-19 mortality, a response vector, **y**, and two sets of matrices (**X**_S_ for static variables and **X**_Y_ for dynamic variables) were compiled. The model was then trained on data from the 366 days in 2020. The model was then tested using a data set consisting of the first 60 days in 2021. Rolling elastic net regression was performed using the linear_model.ElasticNet() function from the scikit-learn-0.24.2 python package. Optimization of the two parameters in elastic net regression, alpha and the l1_ratio, was performed using an exhaustive search method for a random subset of ten countries to minimize the root mean squared error for the predictive model over the test set.

Rolling regression was performed by choosing a minimal window size of 1 day. The training data then consisted of using the mortality data for a single country from the previous day for all countries in conjunction with the dynamic variable values for the given country from the same time window as predictors. The model was trained on the value for 365 days using the previous day as a predictor (the first day in the sequence did not have a preceding date and could not be used in the training set). The window size was then increased to 2 days and calculated each day using the mean and slope of the data within the 2-day window. The model was trained on 364 days, as the first two days in the series did not have a complete set of explanatory variables. The window size was expanded until a size of 60 days was reached, as we were predicting 60 days into the future. A consensus model was then created by averaging the prediction for each model of varying window size.

### Confidence intervals

A bootstrap method was applied to calculate the confidence bounds in the predictions, whereby 20% of the training data was replaced with data sampled randomly from the complete distribution of training data for each given country. The bootstrap procedure was repeated 100 times. The predictions were sorted by value, and the 0.975 and 0.025 quantiles were calculated for each day for the upper and lower bounds of the 95% confidence intervals, respectively. The 0.75 and 0.25 quantiles for the 50% confidence interval were also calculated.

### Data compilation for response variable

In order to train a machine learning algorithm to predict COVID-19 mortality, we compiled the 14-day moving average with two sets of matrices for static and dynamic variables. The model was trained using data from each of the 366 days in 2020 and tested to make predictions on the first 60 days of 2021. The response vector was acquired from the John Hopkins University of Medicine Coronavirus Resource Center (6, 12). Countries were clustered by similarity in dynamics.

### Data compilation for static explanatory variables

To construct the static variable matrix, we searched for global data, by country, for an array of population dynamics to characterize each country’s subsequent mortality due to COVID-19. Preceding the spread of the COVID-19 pandemic, aggregated data relating to a country’s burden of disease (21), comorbidities (16), alcohol consumption (13), proportion population obese (22), access to healthcare (17), economic situation (23), geographic location (6, 12, 19), median age (14), and urbanization (15) were gathered. The Disability-Adjusted Live Years (DALYs) lost per 100,00 population overall and by disease were also compiled (21, 22).

### Data compilation for dynamic explanatory variables

During the expansion of the pandemic in 2020, daily data relating to a country’s climate measures (maximum daily temperature, precipitation, etc.) and non-pharmaceutical interventions (NPI) were collected from the United States NOAA (19) and Oxford University’s COVID-19 Government Response Tracker (18), respectively. To create the matrix of dynamic variables, the data of a country’s maximum daily temperature and precipitation were retrieved for the timeframe of 01 Jan 2020 through 31 Dec 2020 from the NOAA (19). Daily assessments of each country’s Non-Pharmaceutical Interventions (NPI) stringency were collected from Oxford University’s COVID-19 Government Response Tracker (18). The respective government responses included recorded closing of schools and universities, workplaces and public transportation, cancellation of public events and limits on gatherings, shelter in place orders, restriction on international travel, financial support and debt relief, campaigns to educate the population on COVID-19 and safety, and policies on access to COVID-19 testing and facial coverings outside the home.

### Predicting cumulative mortality for 2020

To predict cumulative mortality for each country over 2020 using the matrix of static variables, PLSR was used, which first projects the high-dimensional data into a lower-dimensional space of latent variables. These latent variables represent orthogonal or uncorrelated sources of variance in the data. Only the number of latent dimensions that contributed significant predict power to the model to prevent fitting the model to extraneous noise in the training set were kept.

### Predicting mortality per day using elastic net rolling regression

We incorporated the time dimension to explore how each dynamic variable might contribute to COVID-19 mortality. Each variable may have a different contribution to explaining COVID-19 mortality at a specific lag. The lag is defined as the number of days from infection to the mortality event. To fully utilize the data from dynamic variables, it must be incorporated at a reasonable timeframe before the events a model aims to predict. For every 60 consecutive days in 2020, the parameters of the model including the coefficients, and L1- and L2-penalties were trained to predict the mortality for each of the subsequent 30 days. For validation, the model was then used to predict the mortality for the first 30 days of 2021. Elastic Net Regression (ENR) is a constrained form of Ordinary Least Squares regression whereby an L1 and L2 penalty is applied to the fitted coefficients. The L1- and L2-penalties encourage regression coefficients to remain near zero, thereby preventing overfitting through coefficient inflation.

## Results

**Figure 1:**
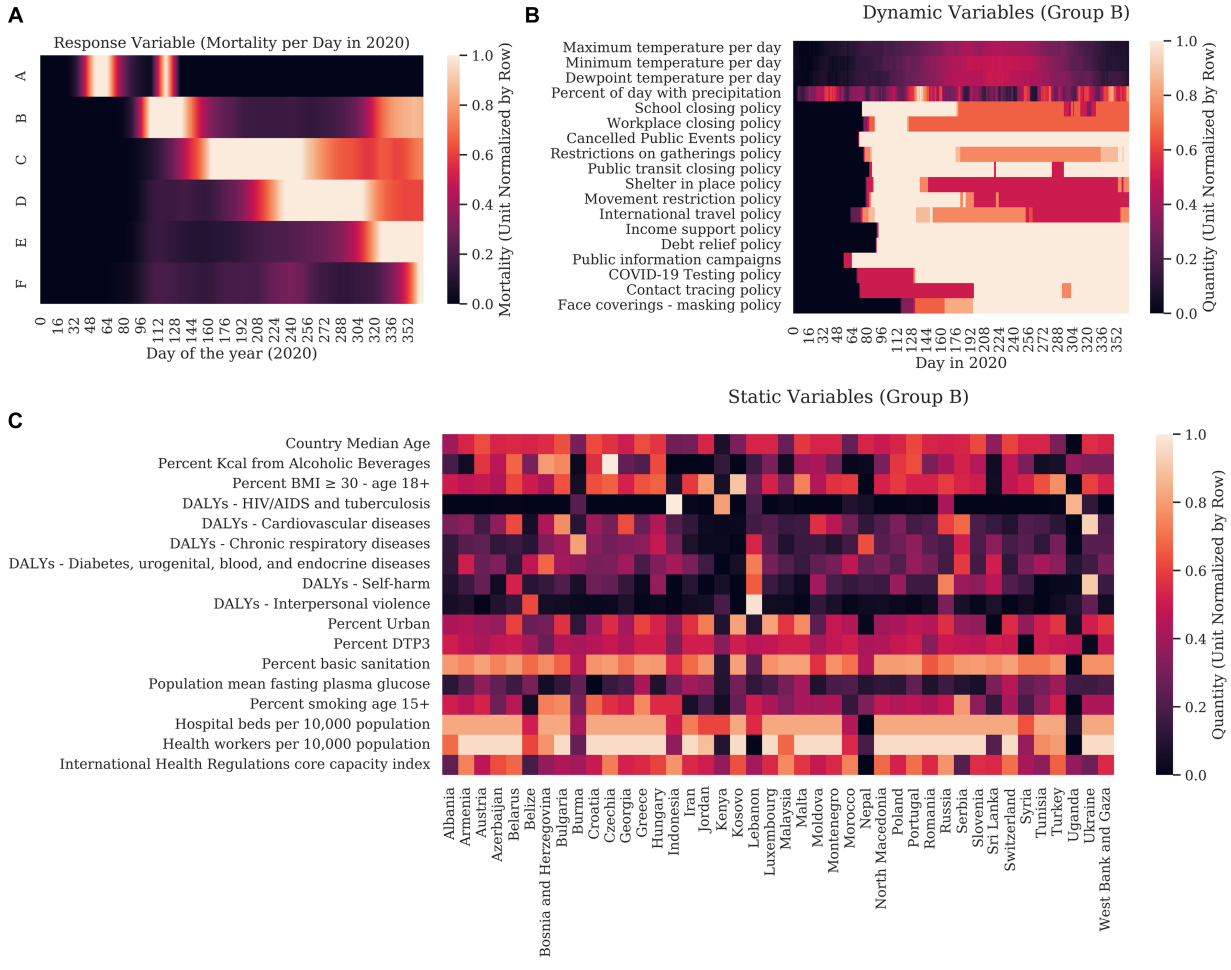
We first graphed the response variable (COVID-19 mortality) for each country for each day in 2020. Columns correspond to days of the year. Hierarchical clustering was used to show how each country related to other countries using cosine similarity. **(A)** For clarity, countries are clustered into groups with similar dynamics with each row representing a mean of the normalized mortality of each country in the group. **(B)** The unit normalized static variables were clustered with the static response variable (the sum mortality for each country over 2020). In this case, rows represent countries and are clustered by cosine similarity. The columns representing the explanatory variables are clustered with the static response variable. **(C)** Graph of the unit normalized explanatory variables for the countries in Group B are shown as an example of how weather and the stringency of governmental interventions varied over the year. Here, columns correspond to the days of 2020 and rows represent magnitude of dynamic variables.

Table 1 Mortality is described in Figure 1A, with countries with similar mortality patterns clustered together in Groups A – F. A complete clustergram of mortality dynamics per country are included in Supplementary Figure S1. To conceptualize the static variables representing countries in Cluster B, the heatmap (Figure 1B) shows the min-max row normalized value for each variable by country. To conceptualize the format of the dynamic variables, we show the dynamics of each variable averaged for the countries in Cluster B in Figure 1C.

For the 17 static variables considered, the 17-dimensional matrix was projected into a lower-dimensional (latent) space. Model error was tested (RMSE) after stepwise discarding the latent dimensions that explained the smallest amount of variance in the response. The scree plot in Supplementary Figure S2 shows the amount of information gained as a function of the number of latent variables included. As most of the information was contained in the first two principal components, the PLSR model was created to predict each country’s cumulative mortality *per capita* in 2020 from the first two principal components of the matrix.

**Figure 2:**
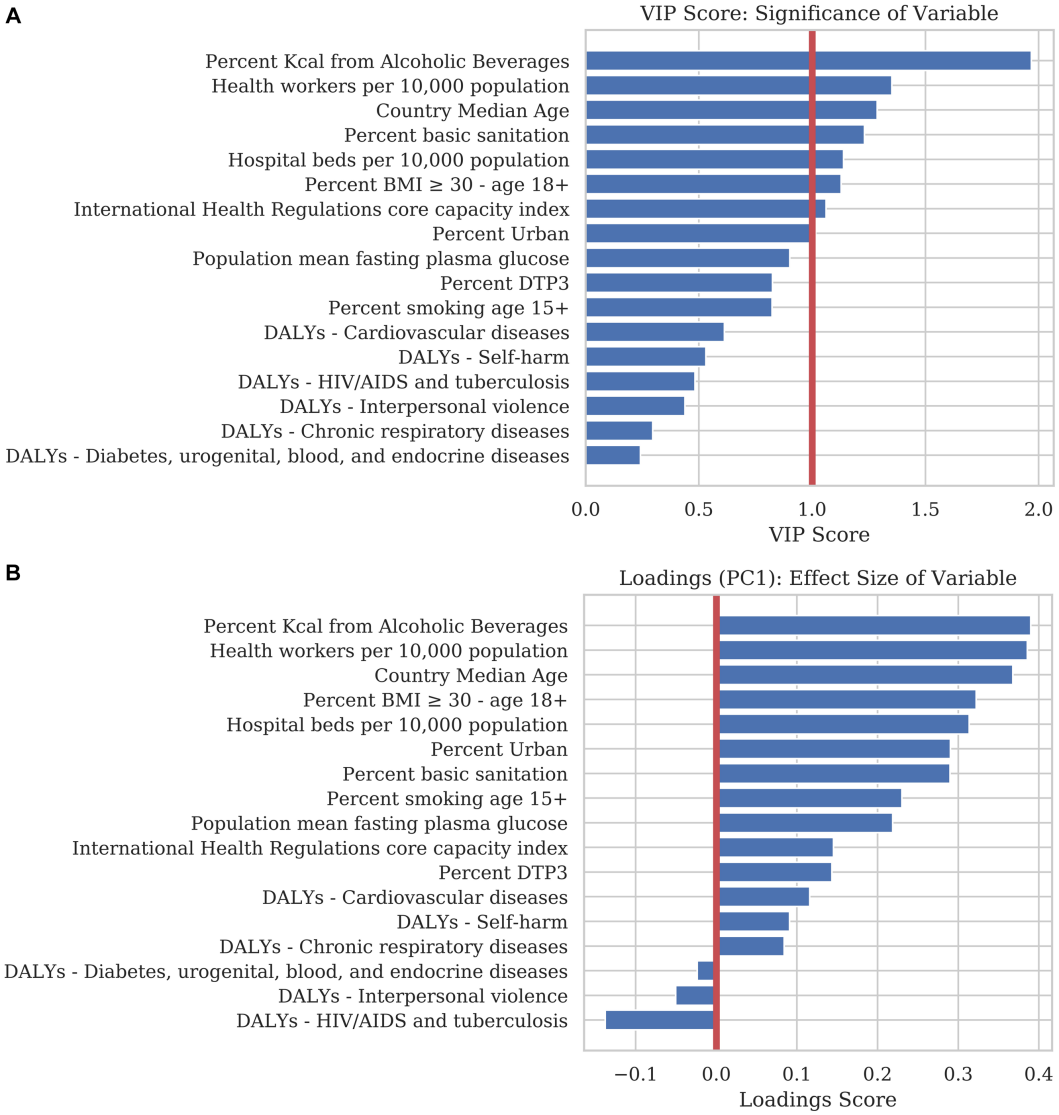
We trained a predictive model using Partial Least Squares Regression (PLSR) to examine the magnitude, directionality, and significance that each explanatory variable contributed to COVID-19 mortality. **(A)** The bar chart is sorted in descending order of the Variance of Importance in Projection (VIP) score. The VIP score is a measure of the significance each variable contributes to the predictive accuracy of the model. Generally, a VIP score below 1.0 can be discarded from a predictive model with little loss in accuracy. **(B)** The loadings of the first principal component (PC1) are an indicator for the magnitude and directionality of the effect of each static variable on explaining mortality. Variables with a positive score correlate positively with mortality whereas variables with a negative score correlate negatively. Loadings close to the zero line indicate that the variable contributes little to the model.

Figure 2A illustrates each static variable’s Variance of Importance in Projection (VIP score). The VIP score measures how much each variable contributes to the projection in two dimensions. This value corresponds to the amount of predictive power that each variable adds to the model. The volume of alcohol consumed by the population had the most predictive power of cumulative mortality in 2020, followed by the number of health workers *per capita* and the median age of the individuals in each country. A threshold of 1.0 is applied to PLSR models to separate significant variables from those that contribute little to the model. Using this threshold, percent kcal from alcoholic beverages, health workers per 10,000 population, country median age, percent basic sanitation, hospital beds per 10,000 population, percent BMI ≥ 30 – age 18+, international health regulations core capacity index, and percent urban reasonably predicted, with positive associations, with the number of COVID-19 deaths expected per country, in the absence of government/public health interventions.

**Figure 3:**
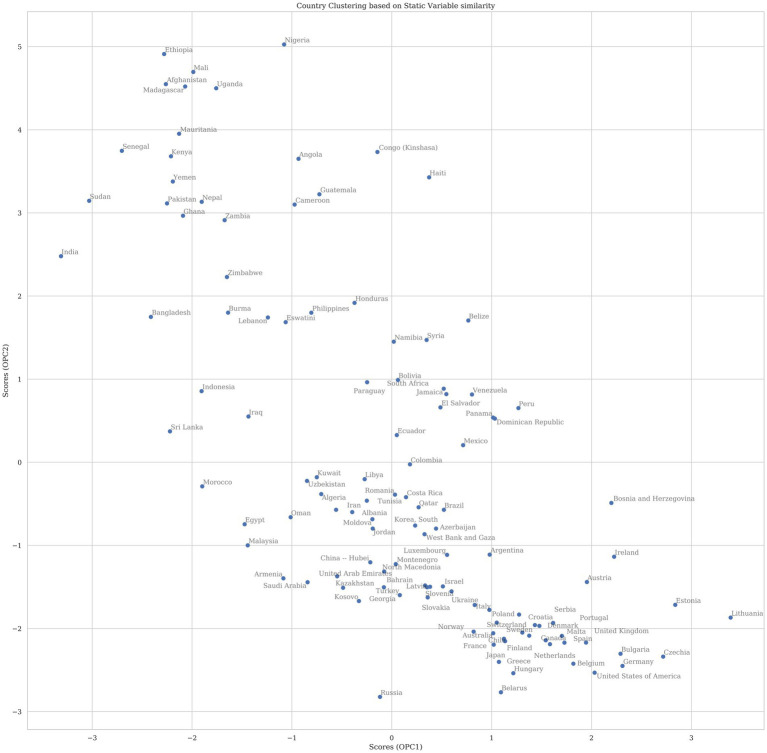
The plot above shows the scores for countries projected into the first two principal components (PC). The components were rotated so that the first PC on the x-axis corresponds to variance that explains COVID-19 mortality whereas the second PC is all other variance in the model. Countries that appear near to each other in two-dimensional space have higher similarity of variables that have explanatory effect on total mortality for 2020 than countries that appear further apart. Countries with a higher x-value are predicted in this model to have higher COVID mortality if dynamic factors were not considered.

The PLSR scores for countries are plotted in Figure 3 in the same latent space. Countries with similar demographics cluster together on the scatterplot. Countries that appear far to the right are predicted to have the highest COVID-19 mortality in 2020, given the demographics listed, while countries to the left are predicted to have low mortality.

In order to evaluate the significance and effect size of the dynamic variables, we summed each dynamic variable for every day in 2020. We then created a PLSR model using the same method developed for the static variables with the summed dynamic data. Examining the scree plot shown in Supplementary Figure S3, we observed that most of the data was contained in four principal components, as opposed to the two in the static variable model. For this reason, we included four principal components (PC) in the dynamic model.

**Figure 4:**
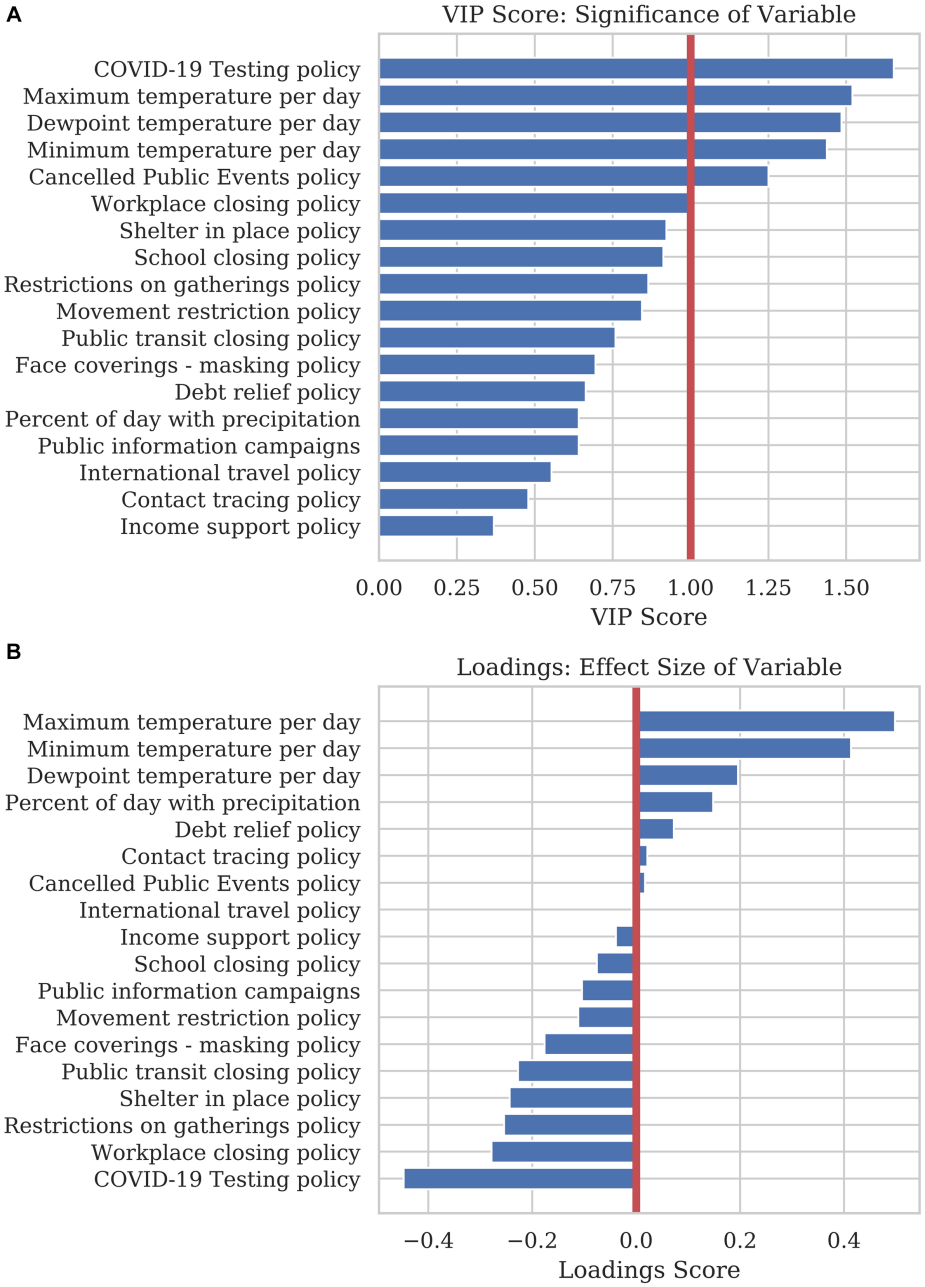
A PLSR model was used to find the significance and effect size of dynamic variables in explaining COVID-19 mortality. **(A)** The bar graph shows the VIP score of each dynamic variable. The VIP score is a measure of how much predictive power each variable adds to the model. **(B)** The loading plot of the first principal component show the directionality and effect size that each variable adds to the prediction. Variables with a positive loading contribute to a higher mortality prediction, whereas variables with a negative loading contribute to a lower prediction for the response variable.

Figure 4A shows the VIP score corresponding to each variable’s significance in adding to the projection. The most parsimonious model could be reduced to six variables: COVID-19 testing policy, the maximum temperature per day, dewpoint temperature per day, minimum temperature per day, canceled public events policy, and workplace closing policy. These variables were evaluated on a daily basis, as mortality accumulated over time. They represent what countries did, or experienced. Other variables may be significant in reducing COVID-19 mortality, but most of the information regarding the predictive model is contained within these six dynamic variables.

The loading plot in Figure 4B shows each variable’s effect size and directionality. Variables with a positive loading score in principal component 1(PC1) positively contribute to mortality, whereas a negative loading score negatively contributes to mortality. As these variables change considerably over the year, this high-level analysis only illustrates how the sum of the variables over the year contributes to the overall mortality for 2020. In order to create a combined model using static and dynamic variables and observe how variables affect mortality based on timing, a model was created to predict mortality per country per day using sliding-window Elastic Net regression.

Rolling regression uses data in a given window of past timepoints to predict future results. To create an accurate model for days immediately in the future, we created a consensus model from the average of many window sizes. To predict the next day into the future, we used a window size of 1, where each of the previous 200 days was used as a predictor. For the second day into the future, we combined the previous model with a new one with a window size of 2. For a window size of 2, we combined the data for two days into the past and used the slope and mean of the window as the predictor. We continued this process until we reached a window size of 60 in order to predict 60 days into the future.

**Figure 5:**
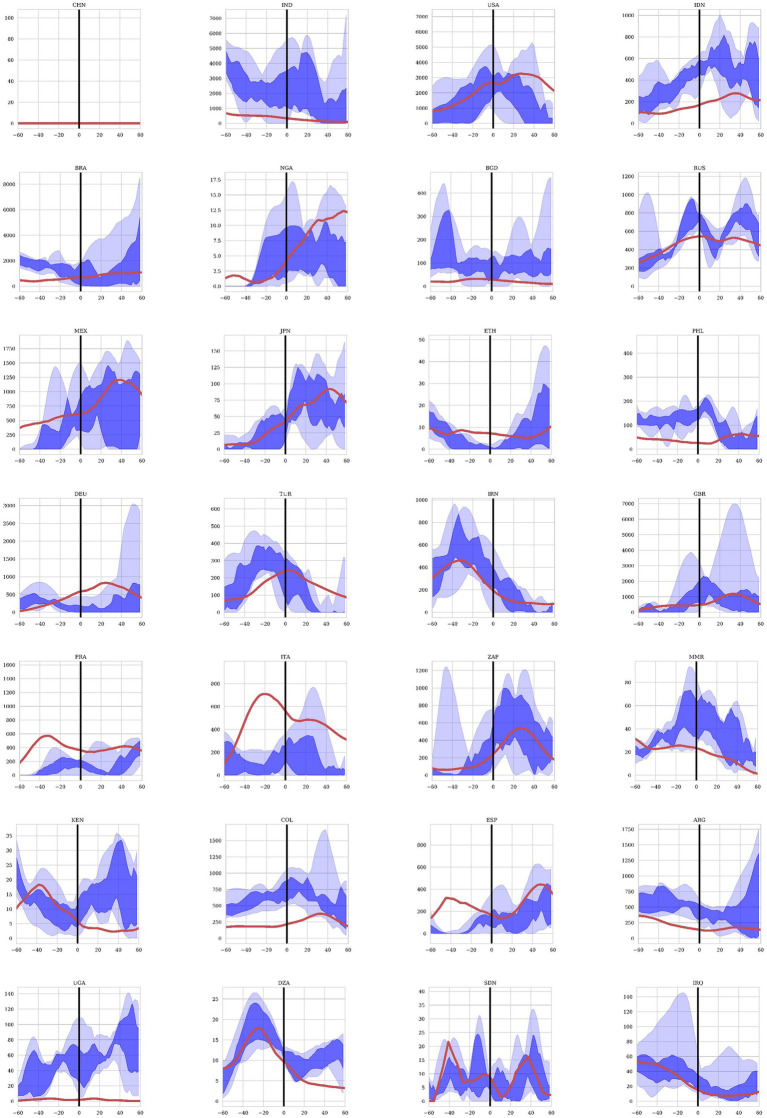
Mortality projections for 28 countries using the Elastic Net regression model. Twenty-eight countries were included in the study in descending order of total population. The projections are for the last 60 days of 2020 representing the training set and the first 60 days of 2021 representing the test set. The dark blue region shows the projections between the 25% and 75% quantiles and the light blue area shows the projected values between the 97.5% and 2.5% quantiles after 100 bootstrapped projections. The red line represents the observed COVID19 mortality as reported by each country.

The predictions from rolling Elastic Net regression are shown in Figure 5 for the 28 most populous countries in the world. The regression included the mortality data per day for every country in the model, as well as the dynamic variables including weather and NPIs for the specific country being predicted. In this way, the model is informed by the country-specific demographics inherent in the past mortality data as well as the dynamic variables that influences how the future mortality time-courses. We calculated the confidence bounds of the predictions by bootstrapping the prediction 100 times. The dark blue region in the figure show the area of the 50% confidence bounds while the wider light blue region shows the area within the 95% confidence bounds. The red line shows the actual mortality data reported for each country over the same point in time. Data was predicted for the last 60 days of 2020 (shown as −60 to 0 days in the figure) to assess the accuracy of predicting with the training time period. The first 60 days of 2021 (shown as 0 to 60 days in Figure 5) were predicted only on data from 2020 to give us an assessment of data on a test set.

**Figure 6:**
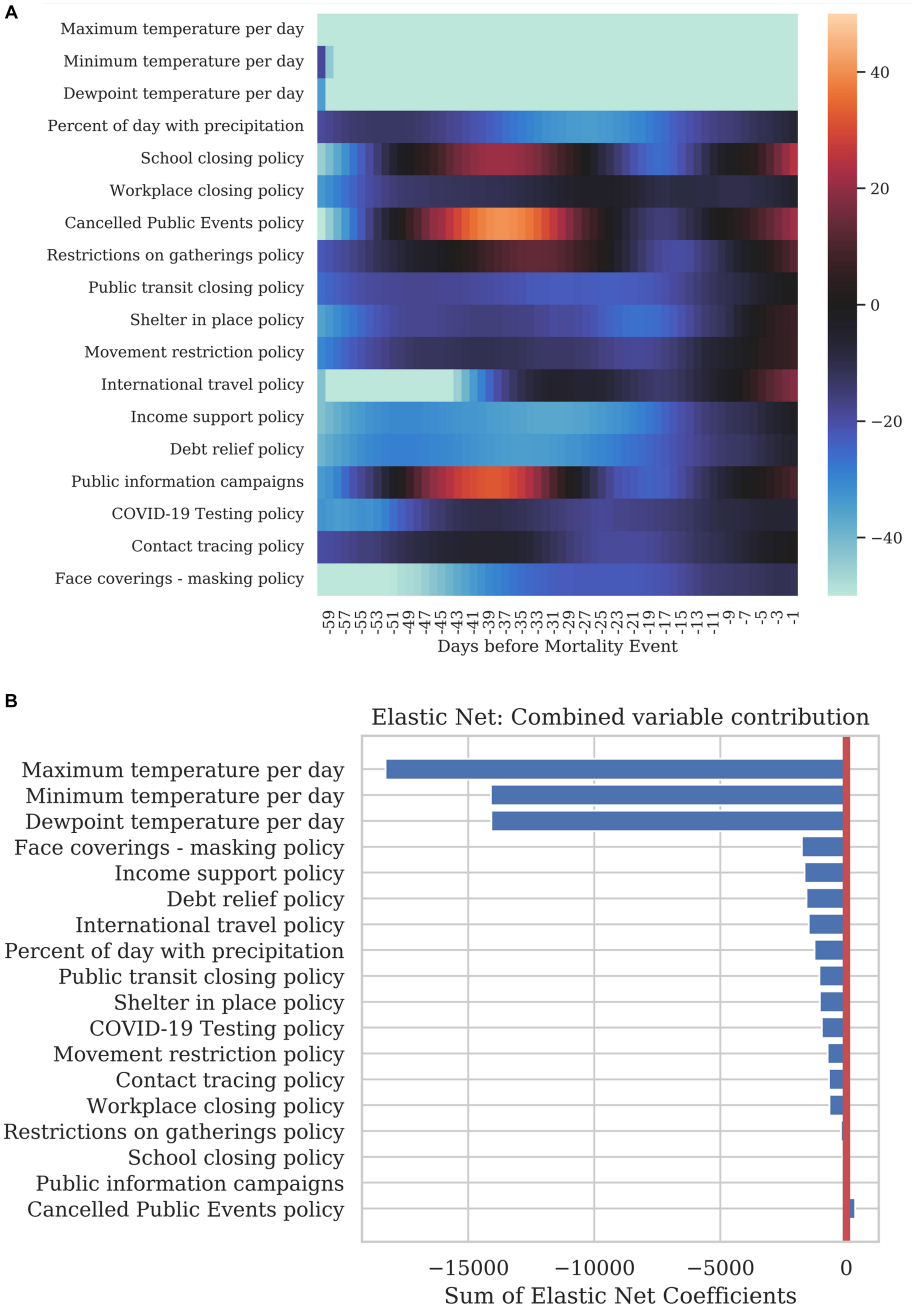
Summary of how each dynamic variable contributed to COVID-19 mortality. **(A)** The heatmap shows the correlation of each variable at specific time before the mortality on the day being predicted. The leftmost column corresponds to a correlation with a 1-day lag and the rightmost column with a 60-day lag. **(B)** The bar graph shows the sum total of all the coefficients per variable for the previous 40 days.

Finally, we examined the value of the regression coefficients from the rolling regression to determine the explanatory power of each variable at specific time points before a mortality event. Figure 6A shows the regression coefficients for the timepoints from 1–60 days before a mortality event. Columns correspond to the lag in days while the rows show each dynamic variable. A blue color at a specific timepoint for a variable is indicative of the variable decreasing mortality for that specific lag. A red color represents an increase in the model prediction for mortality at the specified lag. These coefficients show that some dynamic variables show a sustained decrease in mortality over a wide range of timepoints. Some coefficients have positive or negative influence depending on the amount of time before the mortality event, suggesting that the influence of the variable varies over time. For example, all variables show less of an influence within 7 days of the response as the infection was likely to occur before this time period. Figure 6B shows a simple sum of the coefficients over the 60 days preceding a mortality event.

## Discussion

Understanding and predicting COVID-19 mortality involves considering its multi-factoral nature – pathogen, host, social, environmental in addition to vaccination and treatment factors – with both global and regional components. Traditional prognostic models employed since the beginning of the COVID-19 pandemic have yielded some successes in predicting mortality and the associated surge of infections the population levels but generally in very region-specific contexts and with wide confidence intervals. With the COVID-related data reported waning, and experiencing significant data issues (4), many models have discontinued producing forecasts altogether (5–7). These data quality issues are observed in Figure 5, where forecasted mortality from the machine learning models deviates from reported mortality in certain countries While the prediction of mortality was more accurate for some countries than others, given the disparate methods of quantifying mortality between countries as well as the quality of data integrity, the Elastic-Net model still was able to predict the directionality of trends and reduce the number of predictive variables down to a small subset of significant predictors, lowering the search space for causal factors driving COVID-19 mortality. The machine learning model presented here evaluated multiple factors that contributed to COVID-19 mortality in 2020, before the introduction of anti-COVID-19 vaccinations. By establishing the baseline effect of other variables, the model can also be valuable for predicting COVID-19 mortality in the presence of vaccinations and treatments by accounting for differences in efficacy, availability, and uptake. Some mortality projections for the first 60 days of 2021, before the wide spread availability of vaccinations, are presented here and demonstrate the value of the model for COVID-19 mortality forecast. The projections also offer a glimpse into the application of this machine learning model in the future. Further analyses incorporating data related to vaccinations and therapeutics are necessary to ensure this machine learning model can accurately predict COVID-19 mortality.

Based on the results of the model we observe significant contributions of static variables in predicting mortality. Use of alcohol, health care workers and hospital beds per 10,000 population, the median age of a country’s population, basic sanitation, obesity, urbanization and core capacity index all are useful in understanding a country’s position to manage introduction of a pandemic. In regards to dynamic variables, or variables which move in time with the outcome, climate measures and manageable interventions such as testing policy and calcellation of public events / workplace closings were highly associated with mortality.

As an important note, these variables cannot be considered causal, only predictive. It is also quite possible that a variable serves as a proxy for another factor more clearly within a causal pathway of COVID-19 mortality. For example, number of health workers are highly correlated with median age of a population. To demonstrate this quantitatively, Supplementary Figure S4 shows the PLSR loadings plot which demonstrates how each static variable relates to each other. The loadings plot shows the explanatory variables in the new reduced latent space. Variables closer on the plot have a higher covariance than those that are further apart. To separate the variance related to the response from the unrelated, the latent space was rotated so that all variance related to mortality was parallel to the x-axis in a process known as Orthogonal Partial Least Squares Regression (OPLSR). There are some important additional limitations to the data used for analysis and application of the machine learning model. That causality cannot be determined between the variables and estimated mortality is particularly relevant to COVID-19-related public health policies because the data shows dates of policy introduction rather than when or how well the policy was implemented. That increased number of health workers per 10,000 population, hospital beds per 10,000 population, and percent basic sanitation were associated with increased mortality are counter-intuitive, likely related to other variables (perhaps more clearly on a causal pathway), and an important caveat to blind acceptance that associations derived through machine learning algorithms represent cause-effect associations. Instead, they represent a country’s situation in the year prior to COVID, describing the environment in which the disease spread. Many are not modifiable. They can provide insight to our current societal structures, especially when compared to a century ago, and provide a base upon which to further untangle complex relationships among host, pathogen and environmental conditions.

Each variable which had an impact on COVID-19 mortality had a unique delay from the measurement to the change in COVID-19 mortality. Mortality data is available on a very granular level globally, however it is likely under-reported. Issues of accuracy and consistency in reporting mortality may undermine predictions of future mortality. Global mortality, vaccination, and booster uptake is reported on an aggregated level, with no information regarding age, sex or comorbidities. While these considerations can be understood with more focused analyses on a country or regional level, globally these cannot currently be directly included in mortality forecasts. Similarly, the time period between variable observations (e.g., masking mandates, travel restrictions, etc.) and mortality are nearly always regionally somewhat unique.

## Conclusion

Considering prior models have retired due to data degredation, this machine learning model presents a framework for forecasting mortality for this pandemic, and future pandemics. Responding to the multi-dimensional and shifting nature of the COVID-19 pandemic and its regional variability in both static and dynamic factors, this machine learning method may be a valuble tool for starting to untangle the complex factors which contribute to globally dynamic COVID-19 mortality and provide a framework for investigating influential factors in subsequent pandemics.

## Data availability statement

Publicly available datasets were analyzed in this study. This data can be found here: https://github.com/ciaccmx/covid19ai.

## Author contributions

MC, CS, AP, RF, KC, GK, DH, WK, and LK all participated in the conception and design of this study. MC and CS collected data. MC and AP performed analytic calculations. All authors contributed to the article and approved the submitted version.

## References

[1] Taleghani N Taghipour F. Diagnosis of Covid-19 for controlling the pandemic: a review of the state-of-the-art. Biosens Bioelectron. (2021) 174:112830. doi: 10.1016/j.bios.2020.112830 33339696 PMC7694563

[2] De Wit E Van Doremalen N Falzarano D Munster VJ. Sars and Mers: recent insights into emerging coronaviruses. Nat Rev Microbiol. (2016) 14:523–34. doi: 10.1038/nrmicro.2016.81 27344959 PMC7097822

[3] Mackay IM Arden KE. Mers coronavirus: diagnostics, epidemiology and transmission. Virol J. (2015) 12:222. doi: 10.1186/s12985-015-0439-5 26695637 PMC4687373

[4] Organization WH. Who Coronavirus (Covid-19) Dashboard (2023) (Accessed September 22, 2023). Available at: https://covid19.who.int/data.

[5] Evaluation TIfHMa. Covid-19 Projections (2022) (Accessed September 22, 2023). Available at: https://covid19.healthdata.org/global?view=cumulative-deaths&tab=trend.

[6] Coronarvirus Resource Center, Johns Hopkins University Medicine. (2020). Johns Hopkins University Medicine. Medicine Jhu. https://coronavirus.jhu.edu/map.html (Accessed May 5, 2021)

[7] Prevention USCfDCa. Covid-19 Forecasting (2022) (Accessed September 22, 2023). Available at: https://www.cdc.gov/coronavirus/2019-ncov/covid-data/forecasting-us.html.

[8] Chu DK Akl EA Duda S Solo K Yaacoub S Schunemann HJ. Physical distancing, face masks, and eye protection to prevent person-to-person transmission of Sars-Cov-2 and Covid-19: a systematic review and Meta-analysis. Lancet. (2020) 395:1973–87. doi: 10.1016/S0140-6736(20)31142-9 32497510 PMC7263814

[9] Moulaei K Shanbehzadeh M Mohammadi-Taghiabad Z Kazemi-Arpanahi H. Comparing machine learning algorithms for predicting Covid-19 mortality. BMC Med Inform Decis Mak. (2022) 22:2. doi: 10.1186/s12911-021-01742-0 34983496 PMC8724649

[10] Bottino F Tagliente E Pasquini L Napoli AD Lucignani M Figà-Talamanca L. Covid mortality prediction with machine learning methods: a systematic review and critical appraisal. J Pers Med. (2021) 11:893. doi: 10.3390/jpm11090893 34575670 PMC8467935

[11] Subudhi S Verma A Patel AB Hardin CC Khandekar MJ Lee H. Comparing machine learning algorithms for predicting Icu admission and mortality in Covid-19. NPJ Digit Med. (2021) 4:87. doi: 10.1038/s41746-021-00456-x 34021235 PMC8140139

[12] Time Series Covid-19 Deaths, Global. John Hopkins Whiting School of Engineering; Center for Systems Science and Engineering. https://github.com/cssegisanddata/covid-19/tree/master/csse_covid_19_data/csse_covid_19_time_series (accessed: 05 May 2021).

[13] Covid-19 Healthy Diet Dataset. https://www.kaggle.com/mariaren/covid19-healthy-diet-dataset.

[14] Central Ingelligence Agency. The World Factbook: Country Comparisons – Median Age. https://www.cia.gov/the-world-factbook/field/median-age/country-comparison. (Accessed May 13, 2021).

[15] World Bank. United Nations Population Division. World Urbanization Prospects: (2018). Available at: https://data.worldbank.org/indicator/sp.urb.totl.in.zs.

[16] Our World in Data. Burden of disease by cause, WORLD, (2017). Available at: https://ourworldindata.org/grapher/burden-of-disease-by-cause?country=~owid_wrl.

[17] Who. Primary Health Care on the Road to Universal Health Coverage: (2019) Monitoring Report 2019. Available at: https://www.who.int/docs/default-source/documents/2019-uhc-report.pdf.

[18] Hale T Angrist N Goldszmidt R Kira B Petherick A Phillips T. A global panel database of pandemic policies (Oxford Covid-19 government response tracker). Nat Hum Behav. (2021) 5:529–38. doi: 10.1038/s41562-021-01079-8 33686204

[19] National Oceanic and Atmospheric Administration. Integrated Surface Data (2020) [3/15/2021]. Available at: https://www.ncei.noaa.gov/data/global-hourly/access/2020/.

[20] Greenacre M Groenen PJF Hastie T D’Enza AI Markos A Tuzhilina E. Principal component analysis. Nat Rev Methods Prim. (2022) 2:100. doi: 10.1038/s43586-022-00184-w

[21] Esteban Ortiz-Ospina and Max Roser (2016) – "Global Health". Published Online at Ourworldindata.Org. Available at: https://ourworldindata.org/health-meta [Online Resource].

[22] World Health Organization. Global Health Observatory Data Repository. Prevalence of Obesity among Adults, Bmi ≥ 30, Age-Standardized Estimates by Country. Available at Https://Apps.Who.Int/Gho/Data/Node.Main.A900a?Lang=En (Accessed June 19, 2021).

[23] Gross National Income, Expressed in Current International Dollars Converted by Purchasing Power Parity Conversion Factor. International Comparison Program, World Bank. World Development Indicators Database. Eurostat-Oecd Ppp Programme. Last Updated: Jun 30, 2021. https://data.worldbank.org/indicator/ny.gnp.pcap.pp.cd?view=chart.

