# Supplementary figures and images for: A time-course prediction model of global COVID-19 mortality
Source: Front Public Health. 2023 Dec 7;11:1232531. doi: 10.3389/fpubh.2023.1232531 (PMC10773778; doi:10.3389/fpubh.2023.1232531)

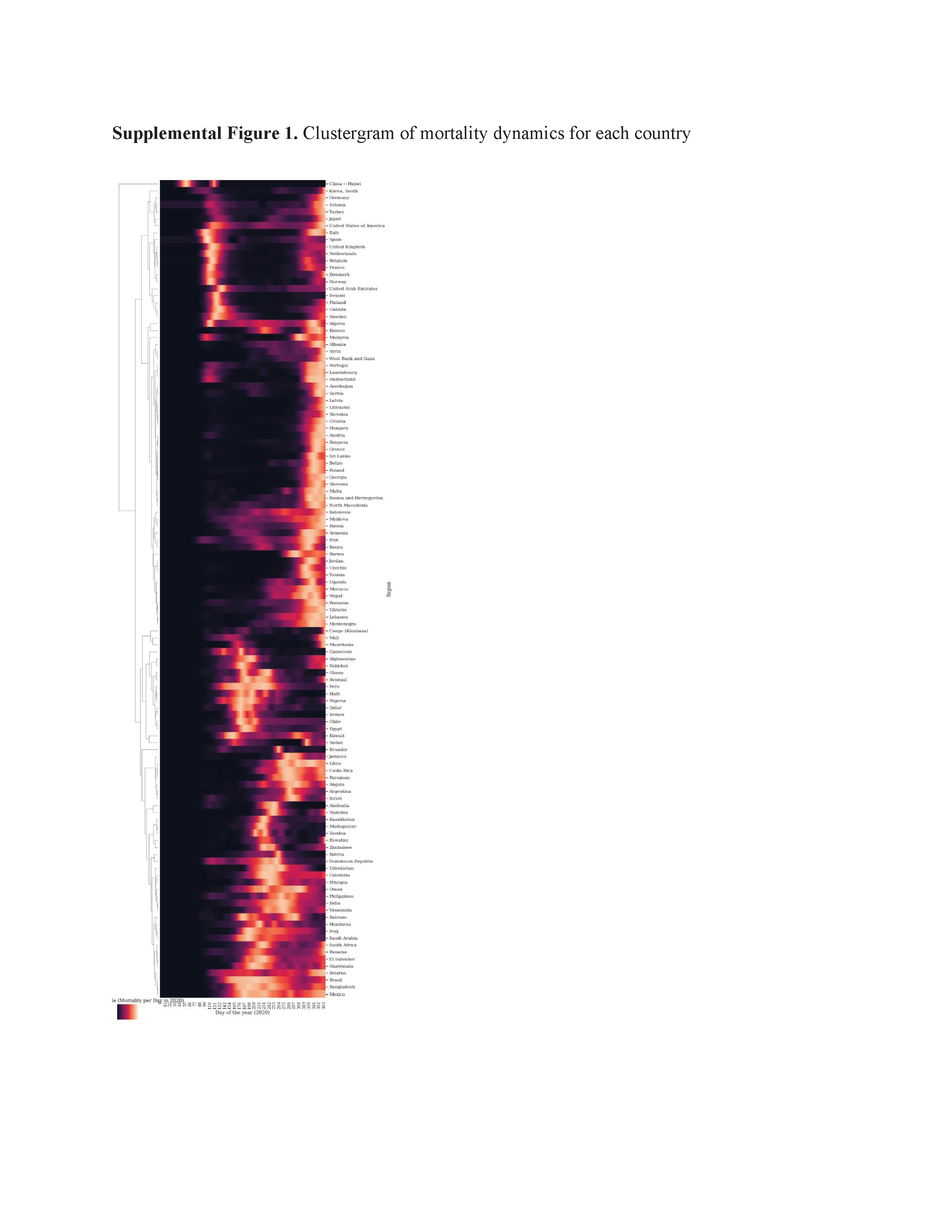

Supplement: Supplementary file 1 [file Image_1.JPEG]

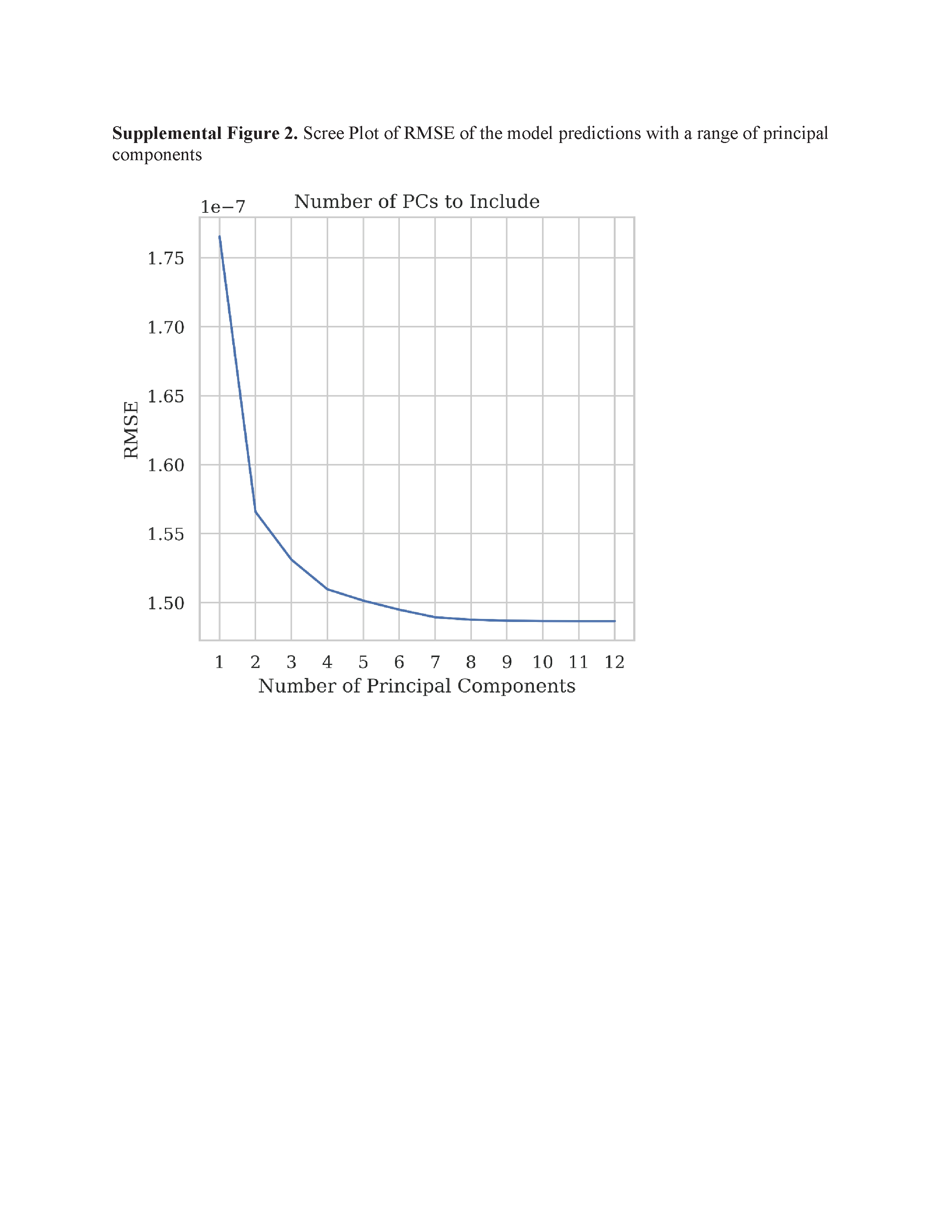

Supplement: Supplementary file 2 [file Image_2.JPEG]

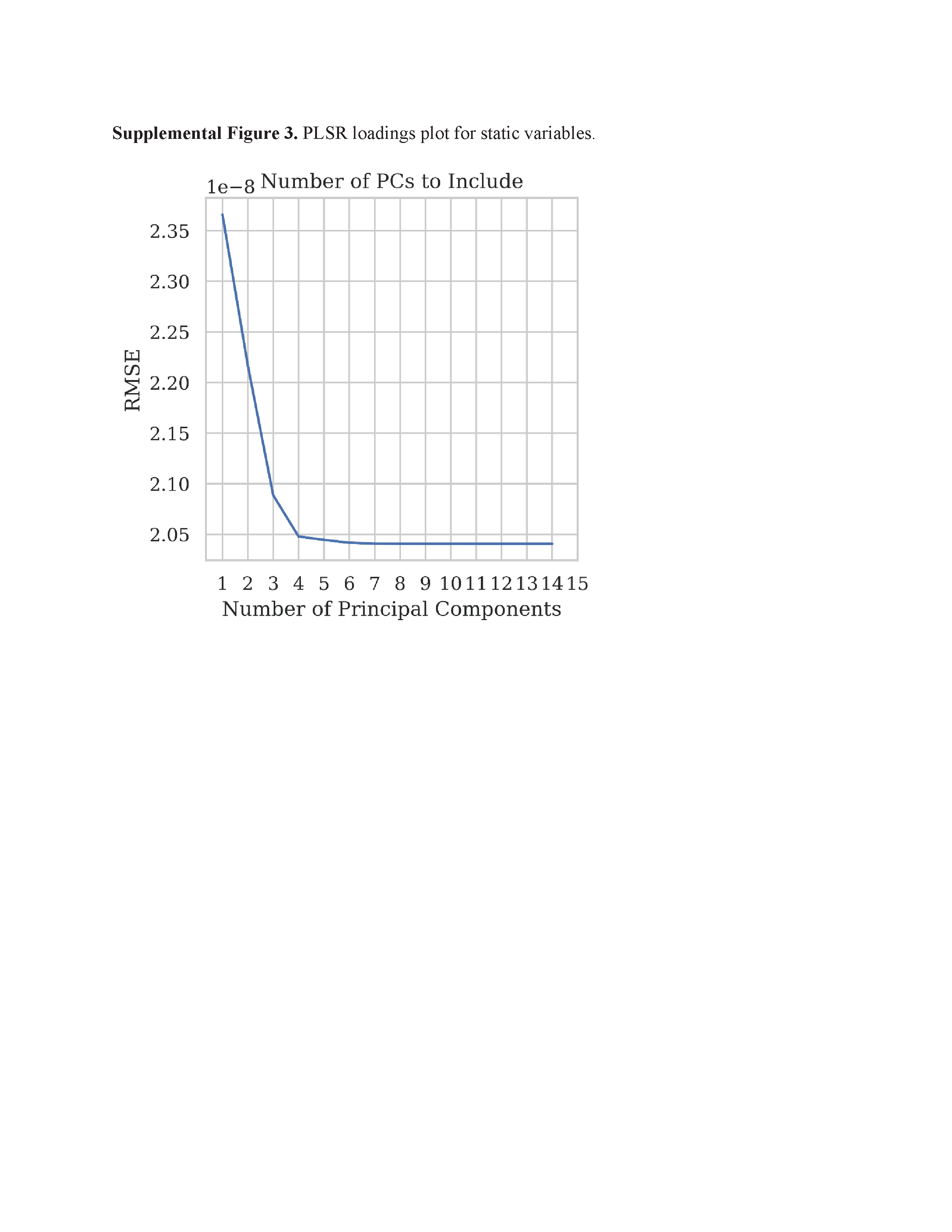

Supplement: Supplementary file 3 [file Image_3.JPEG]

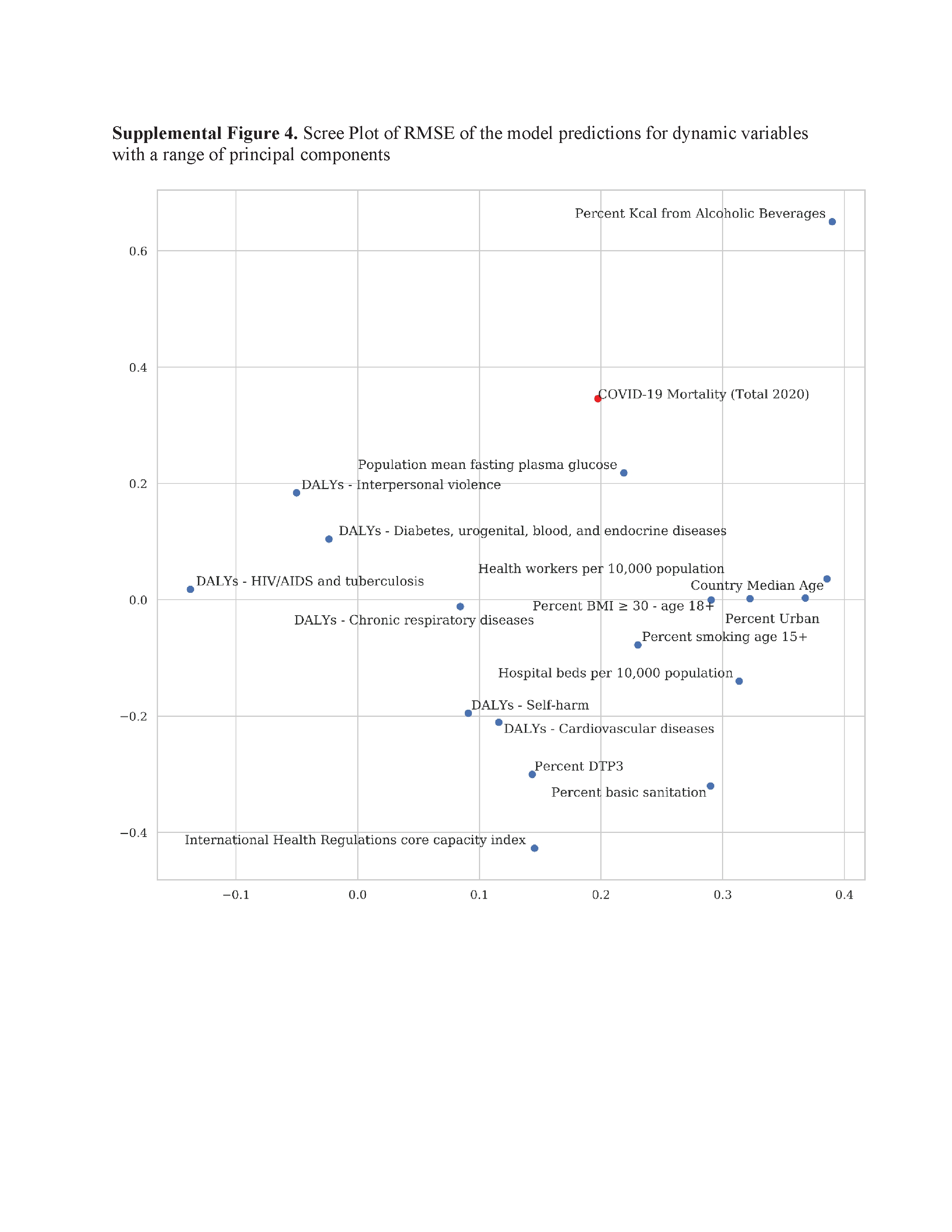

Supplement: Supplementary file 4 [file Image_4.JPEG]
